# The roles of chaplains in dispelling cancer myths in Nigeria: A narrative review

**DOI:** 10.1002/hsr2.1502

**Published:** 2023-08-21

**Authors:** Afeez A. Salami, Kehinde K. Kanmodi, Jimoh Amzat

**Affiliations:** ^1^ Department of Oral and Maxillofacial Surgery University College Hospital Ibadan Nigeria; ^2^ Faculty of Dentistry University of Puthisastra Phnom Penh Cambodia; ^3^ Cephas Health Research Initiative Inc Ibadan Nigeria; ^4^ School of Dentistry University of Rwanda Kigali Rwanda; ^5^ School of Health and Life Sciences Teesside University Middlesbrough UK; ^6^ Department of Sociology Usmanu Danfodiyo University Sokoto Nigeria; ^7^ Department of Sociology University of Johannesburg Johannesburg South Africa

**Keywords:** cancer, chaplains, control, misconception, myths, narrative review, Nigeria, prevention, public health, religious leaders

## Abstract

**Background and Aims:**

The epidemiological burden of cancer in Africa, and Nigeria in particular, has been increasing significantly over the past few decades due to exposure to numerous risk factors as well as belief in various myths and misconceptions. Chaplains can play crucial roles in dispelling these myths and misconceptions about cancer among people. Therefore, this study seeks to review the epidemiological burden, risk factors, and myths relating to cancer and the roles of chaplains in dispelling cancer myths in Nigeria.

**Methods:**

This paper is a narrative review that relied on secondary sources obtained through a thorough literature search of relevant articles on multiple electronic databases including PubMed, Google Scholar, and Web of Science. Published books, journal articles, and other published materials that were written in English were consulted in line with the objectives of this study. Both theoretical and empirical papers were used for this review.

**Results:**

Cancers are associated with risk factors including exposure to chemicals, ultraviolet radiation, harmful tobacco and alcohol use, exposure to human papillomavirus (HPV), and these factors may vary with age, cultural beliefs (myths and misconceptions), and socioeconomic factors among others. Chaplains, however, have crucial roles to play in dispelling cancer myths in Nigeria. These roles include counseling, advocacy, education, and psycho‐social support which may be limited by challenges such as spiritual ambiguity, inadequate training of healthcare providers and limited time/resources. These challenges can be addressed by training healthcare providers and incorporating chaplain practice in Nigerian healthcare.

**Conclusion:**

The role of chaplains in dispelling cancer myths in Nigeria is crucial despite the numerous challenges. Hence, an urgent address of these challenges will be instrumental in ensuring effective chaplain practice in Nigeria.

## 1 INTRODUCTION

Cancers are a group of diseases characterized by rapid and abnormal growth of cells with the potential for local invasion and distant spread to other tissues of the body.^1^ Globally, cancer remains one of the leading causes of death and its burden has been growing rapidly.^2^ One in five men or one in eight women worldwide will develop cancer during their lifetime. Similarly, 1 in 8 men or 1 in 11 women will die from the disease.^3^

In 2020 alone, over 19 million new cases of cancer and about 10 million cancer‐related deaths were recorded worldwide with the highest fatality rates seen in Asia and Africa.^3^ In addition, female breast cancer is the most diagnosed cancer type; closely followed by lung cancer, the commonest type of cancer in men and the leading cause of cancer‐related deaths worldwide.^4^

Africa contributes to over one million cancer burden and over 700,000 cancer mortalities and this trend will continue to rise due to population growth, harmful alcohol and tobacco intake, risky sexual practices, obesity, and so forth.^5^

In Nigeria, reports indicate that over 100,000 new cancer cases and over 70,000 cancer deaths occur annually.^6^ Despite this growing burden, cancer has received low public health priority partly due to a lack of appropriate cancer policies, lack of regulation of cancer known risk factors, limited cancer literacy, insufficient funding, inadequate screening programs, uncoordinated cancer registry/data,^7^, ^8^, ^9^ and so forth. Also, a diagnosis of cancer in Nigeria is seen as a death penalty due to various myths and misconceptions among the people. Myths represent historic and symbolic oral stories that interpret events, realities, and belief of a people which is transmitted from one generation to the other. In Africa, myths explain the relationship of humans with their divine deities and express the fact that human misfortunes are a result of disobedience to divine commands and moral codes of their deities. Hence, diseases including cancers are judged as a form of punishment from the supreme beings to humans^10^ and this may explain the various myths and misconceptions about cancer in Nigeria.

Religious leaders including chaplains have a significant influence on people's behaviors including disease prevention. They are leaders and role models capable of championing health messages to people including the vulnerable groups who may be difficult to reach through support, coordination, and mobilization for appropriate change in health behavior.^11^ In addition, chaplains offer spiritual care in healthcare institutions as well as teaching, public education/advocacy, and social support, especially in critical care situations.^12^, ^13^, ^14^ With these in mind, chaplains remain instrumental in dispelling cancer myths and misconceptions globally.

This paper aims to review the epidemiological burden and etiology/risk factors of cancers in Nigeria as well as the roles of chaplains in dispelling cancer myths in Nigeria. The content of this review will provide an in‐depth understanding of Nigeria concerning its current cancer burden status and also provide insights on how to enhance the roles of chaplains in dispelling superstitions about cancers in Nigeria.

## 2 METHODS

**Figure 1:**
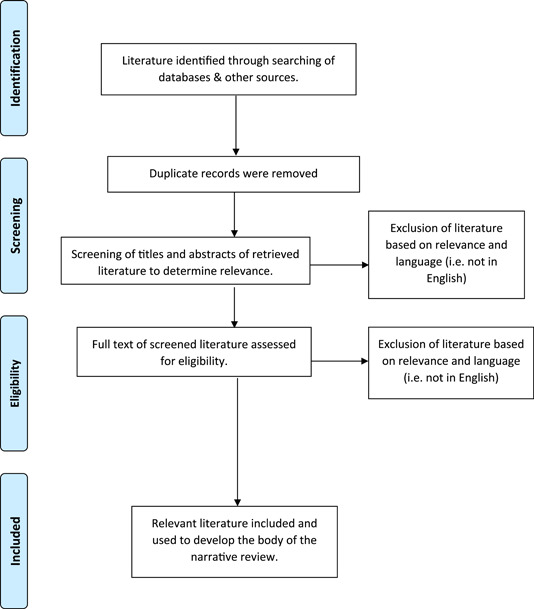
Schematic flow of how the literature used in this narrative review were obtained.

This narrative review relies on secondary sources obtained through a thorough search of relevant literature which was done between January 10, 2023 and March 24, 2023, using multiple search terms, including “chaplains,” “myths,” “misconceptions,” “religious leaders,” “cancer,” “prevention,” “control,” “public health,” “clinical,” “challenges,” “issues,” “recommendations,” and “Nigeria” on multiple electronic databases including PubMed, Google Scholar, and Web of Science. Published books, journal articles, and other published materials that were written in English were consulted in line with the objectives of this study. Both theoretical and empirical articles relevant to the scope of this review were utilized. Figure 1 shows the schematic flow of how the articles used in this narrative review were obtained.

## 3 EPIDEMIOLOGICAL BURDEN OF CANCER IN NIGERIA

The cancer burden has continued to rise in sub‐Saharan Africa but its incidence has been greatly underreported in many African countries because of very weak cancer data management in many of these countries.^15^

In Nigeria, the Nigerian National System of Cancer Registries (NSCR) was set up in 2009 to raise national consciousness in cancer registration, set up cancer registries, and revive redundant cancer registries. Today, there are 10 Population‐based Cancer Registries (PBCR) and 20 Hospital Based Cancer Registries (HBCRs) in Nigeria.^16^

The Ibadan Population‐based Cancer Registry (IBCR) established in 1962 and located in the University College Hospital (UCH), Ibadan, Southwest, Nigeria is the oldest cancer registry in Nigeria and has been publishing cancer data since the early 1960s though its outputs have been inconsistent.^17^ It obtains data from Ibadan and neighboring rural and urban areas. Other population‐based cancer registries include the Calabar Population‐Based Cancer Registry established in 1979, Abuja Population‐Based Cancer Registry established in 2009, Enugu Cancer Registry which was set up in 1988 among others.

Also, the Abuja Population‐based Cancer Registry (ABCR) is located in the National Hospital, Abuja (North‐central part of Nigeria), and was established in 2009 as a population‐based cancer registry. The registry actively obtains data from all the departments of the hospital involved in the diagnosis and treatment of cancer patients and from other notification sources including general hospitals and pathology laboratories outside the hospital.

Reports from these two population‐based cancer registries in Nigeria (i.e., IBCR and ABCR) showed a significant increase in cancer incidence in Nigeria over the past few decades. Notably, the age‐standardized incidence rate of breast cancer increased from 13.7 per 100,000 between 1960 and 1969 to 54.3 per 100,000 between 2009 and 2010. This represents about a 200% increase within that period. This increase was however found to be due to improved diagnosis, better case finding, and improved access to care, increasing risk factors among others.^18^ According to recent statistics, about 125,000 new cancer cases and close to 80,000 cancer‐related deaths have been recorded in Nigeria.^6^ The report also revealed that Nigerian women are more predisposed to cancer than their male counterparts (1.4:1).^6^ Breast (38.7%) and cervical cancer (16.4%) are the commonest types of cancer in women while prostate cancer (29.8%) and colorectal cancers (8.4%) are the commonest in men with the average age at diagnosis of most cancers around the sixth decade in Men and fifth decade in women.^6^ Other common cancers among Nigerian Men include non‐Hodgkin lymphoma (7.3%), liver (6.9%), leukemia (3.7%), while non‐Hodgkin lymphoma (4.8%), ovarian cancer (4.4%), colorectal carcinoma (4.3%) are the other common cancers seen among Nigerian women.^6^

## 4 ETIOLOGICAL/RISK FACTORS OF CANCERS IN NIGERIA

The etiological factors of cancer are complex and are associated with important risk factors including age, exposure to chemicals or ultraviolet radiation, harmful tobacco and alcohol use, exposure to human papillomavirus (HPV), genetic predisposition, and so forth.^19^ These risk factors also vary with race, environmental characteristics, cultural beliefs, socioeconomic factors, and cancer literacy, among others.^3^

### 4.1 Tobacco use

Tobacco is highly carcinogenic to humans and is capable of inducing cancerous growth through carcinogens like tobacco‐specific nitrosamines 4‐(methylnitrosamino)−1‐(3‐pyridyl)−1‐butanone and other volatile organic compounds released when tobacco is burned.^20^

Globally, over 20% of death is related to tobacco use and this trend has continued to grow in many countries of the world including Nigeria.^21^ Smoking is associated with at least 16 cancer types including lung cancer, oral/oropharyngeal cancer, laryngeal cancer, liver cancer, stomach cancer, bladder cancer, pancreatic cancer, esophageal cancer, renal cancer, cancer of the paranasal sinuses, and so forth.^22^ It is also associated with childhood leukemia especially Acute myeloid leukemia (AML), hepatoblastoma, and so forth, in children born to parents who smoke.^23^, ^24^ In Nigeria, tobacco smoking, chewing and other forms of tobacco use are risk factors for cancers of the lung, oral cavity, cervix, bladder, larynx, and esophagus.^25^

### 4.2 Alcohol consumption

The metabolism of alcohol in the body produces acetaldehyde as a by‐product which is highly carcinogenic by preventing DNA repair.^26^ Studies have shown that alcohol consumption is associated with cancer of the breast, mouth and pharynx, stomach, larynx, pancreas, and colon.^27^, ^28^ In Nigeria however, harmful alcohol use has been associated with cancers of the liver, breast, oral cavity/oropharynx, gallbladder, pancreas, colon, and esophagus. In addition, breast cancer is the commonest alcohol‐associated cancer in women while colorectal cancer is the most common alcohol‐associated cancer in men. Interestingly, the risk of developing thyroid cancer decreases with mild to moderate alcohol consumption.^29^

### 4.3 HPV

HPV infection is associated with cancers of the anus, vulva, vagina, oral cavity and oropharynx, uterine cervix, penis, and so forth.^30^

Though the causative mechanism remains unclear, reports about HPV‐induced (especially HPV‐16 and HPV‐18 subtypes) cancers suggest a degradation of the tumor suppressor genes, p53 and pRb leading to uncontrolled DNA replication and neoplastic transformation.^31^

The E6 and E7 oncogenes which target p53 and Rb genes respectively are very important in tumorigenesis. The E6 promotes genomic instability while E7 establishes cell immortalization which leads to malignant transformation.^31^ In Nigeria, only about 2% of all cancers in men and 17% of all cancers in women are HPV‐related. This includes cancer of the cervix, anus, vulva and vagina, oral cavity/oropharynx, anus, and penis.^32^

### 4.4 Exposure to chemicals and ultraviolet radiation

Solar ultraviolet radiation (SUVR) has been implicated in the development of various forms of skin cancers including basal cell carcinoma, squamous cell carcinoma, and melanomas, especially in high solar irradiance areas, sun‐sensitive people, and sun‐exposed body sites. The highest rate of skin cancer is recorded in Australia/New Zealand followed by North America and Europe. The highest rate of skin cancer in Africa has been found among South Africans.^33^ In a study conducted among Nigerians, it was reported that albinism is one of the commonest causes of cutaneous malignancies.^34^

Similarly, long‐term exposure to chemicals like pesticides from environmental pollution has been associated with different forms of cancers. For example, chronic exposure to dichlorodiphenyltrichloroethane (DDT) before puberty in girls can be associated with the development of breast cancer later in life.^35^ Many childhood malignancies are associated with parental exposure to chemicals before conception, in‐utero or direct exposure during the early childhood period.^36^ In Nigeria, head and neck malignancies are associated with exposure to agrochemicals, fumes from indoor cooking, and so forth.^37^

### 4.5 Socioeconomic status (SES)

SES is measured by income, education, or occupational status and it is an important predictor of health‐seeking behavior of individuals.^38^ A significant socioeconomic inequality in the incidence, diagnosis, and management of cancers as well as possible survival and rehabilitation of patients with cancer has been previously elucidated. Individuals living in economically deprived areas and those who have low education and income have higher incidence and mortality rates for cancers than those in high socioeconomic class.^39^ SES may also predict the anatomic sites of cancer. Reports showed that cancers of the lungs, colon, oral cavity/oropharynx, cervix, stomach, and liver are associated with low SES partly due to exposure to lifestyle habits like smoking, poor diets, exposure to HPV, hepatitis B and C, Helicobacter pylori, and so forth^39^, ^40^ while breast cancer, skin cancer, and thyroid cancers are associated with high SES.^39^, ^41^, ^42^

In Nigeria, a study by Azubuike et al. showed that high SES is associated with a reduction in the risk for oral and breast cancers.^43^, ^44^

### 4.6 Age

Age is a well‐studied cancer risk factor and the incidence of cancer increases with advancing age.^45^ According to the National Cancer Institute, the incidence rate for cancer climbs steadily as age increases. The data from the NCI's Surveillance, Epidemiology and End Results (SEER) program showed that the median age for cancer diagnosis is 66 years. The incidence rate of cancer increases from about 25 cases per 100,000 people in persons within the 1st and 2nd decade of life to about 350 cases per 100,000 people among those below the age of 50 and over 1000 cases per 100,000 people in persons above the age of 60 years.^46^ This is similar to reports from Nigeria.^44^

## 5 COMMON MYTHS ON CANCERS IN NIGERIA

Cancer has been associated with different myths and misconceptions. It is thought that cancers are the result of past sins, wizardry/witchcraft, evil eye, evil arrows, curse, envy, and forth. It is also believed that cancer results from injuries that can be transmissible/inherited and spread by cutting through it.^47^ These myths and misconceptions vary across countries in Africa and present significant barriers to cancer control as cancer risk prevention lifestyle changes, screening, and treatment are ignored.^48^

The reports from different African countries have indicated that religion and sociocultural beliefs contribute immensely to the myths and misconceptions about diseases in general^49^, ^50^ and cancer in particular.^51^ Notably, a study among female undergraduate students in Cameroon revealed that about 35% of participants cited witchcraft, spiritual attack, God's curse or enemy attack as risk factors for breast cancer. This is in line with reports from female medical students in Ethiopia^51^ as well as reports among rural women in Ghana^52^ and market women from Nigeria.^53^

In addition, a study from Ethiopia showed that cervical cancer was due to breaching social taboos (e.g., a woman exposing her underwear in the sun; instead of an ideal dark position in the house); or undertaking unacceptable behavior. So, women who got ill with symptoms of cervical cancer were excluded from society, received poor social support, and experienced delays in seeking treatment.^54^

In Nigeria however, studies have shown that a significant proportion of patients with cancer believed that cancer emanates from the evil arrow, the type of food they eat, wizardry, multiple sexual partners, and so forth, and this leads to delays in seeking orthodox treatment.^55^ Evil arrow is the attack of the enemy in a supernatural version as a result of witchcraft or wizardry and some communities believe that public naming and shaming of the perceived witches may remedy the ill‐health of the bewitched person.^56^

Furthermore, a lot of people fail to undergo screening for cancer especially cervical cancer screening due to conflicting perceptions they have about the procedure. They believe that once a woman is not promiscuous or does not engage in multiple sexual activities, they are free from having cancer. Hence, the need for not undertaking screening. They also cited individual cultural norms as another barrier to undergoing screening.^57^

In addition, some people in the northern part of Nigeria believe that cancers originate from mystical forces from the forests called *jeji* or *daji* in the Hausa language (the predominant language in the region). Therefore, exposure to these mystical forces leaves one with *jeji* or *daji* which can only be cured through prayers from religious leaders or through the use of herbs from traditional healers/spiritualists. Some communities in the North frown at the mention of the word *jeji* or *daji* for fear of offending the mystical forces and becoming the next victim to get inflicted with *jeji* or *daj*i. Therefore, patients with cancer are compelled to take the option of traditional or religious care for their therapy due to this belief. They visit religious healers (mallams) or local traditional healers/spiritualists known as *Boka* for remedies. They also feared that hospital care worsens the disease. Hence, herbs and prayers are offered for a cure.^58^ This belief system is deeply rooted so much so that elites in this region promote such misconceptions. A very good example is the suspension of all forms of immunization activities from July 2003 to April 2004 because it was believed that polio vaccines contained antifertility components capable of sterilizing their females to depopulate the North.^58^

The view of some religious leaders on cancer generated diverse opinions according to a report from Badru et al.,^59^ on the opinion of religious leaders and seminarians in Nigeria on the causes of cancer. They reported that about a quarter of the respondents believed that sin was responsible for the cause of cancer.

In summary, myths contribute to the underutilization of cancer prevention and care practice among people, and increasing public awareness about them is very pertinent.

## 6 ROLES OF CHAPLAINS IN DISPELLING CANCER MYTHS IN NIGERIA

Cancer imposes fear and uncertainties on patients and their families^60^ and people adapt to these situations with the help of chaplains or spiritual leaders. Though there is a lack of consensus on what constitutes spirituality and chaplaincy in literature, authors have provided some explanations for understanding. While spirituality defines an individual's beliefs, values, behaviors, and experiences which guide their search for life meaning without necessarily involving religion, especially during crises,^61^ chaplains are trained ministers, priests, imams, or representatives of religion attached to a secular organization like the military, hospitals, prisons, schools, and so forth, to provide spiritual care through counseling, teaching, prayers, social support, community organization, and so forth, to those in need and those in some forms of distress.^62^

Cancer myths abound and the duty of dispelling them may be placed on the shoulders of chaplains. Chaplains provide insights into concerns like end‐of‐life issues, family counseling, religious and cultural accommodation as well as provide supportive spiritual care.^62^ Chaplain practice is nonexistent in most Nigerian hospitals and only the church‐owned hospitals provide some form of pastoral care services or spiritual care through visitations and offer of prayers to patients in their hospitals due to lack of professional chaplains.^63^ Therefore, the majority of patients in need of spiritual care will have to rely on the services of nurses (as major caregivers) and sometimes that of their relatives, friends, and members of their spiritual denomination.^64^ Hence, an objective evaluation of spiritual assessment needs of patients becomes very impracticable in Nigeria. Despite these challenges, chaplains have roles to play in dispelling myths associated with cancer in Nigeria and their incorporation into the Nigeria healthcare system will be very crucial. These roles may be summarized below;

1. *Counseling*: From the available evidence, chaplains traditionally focus on addressing the spiritual needs of patients and their loved ones.^65^ This role has however been expanded and has involved ensuring patient safety and counseling among others through active listening, especially when patients are diagnosed with life‐threatening conditions, like cancers.^66^ Healthcare chaplains have also been previously utilized in genetic counseling in situations like genetic/congenital anomalies, miscarriages, abortions, and so forth.^67^ Given these, chaplains will be instrumental in the counseling and motivation of patients against cancer myths and misconceptions in Nigeria. This will promote early cancer screening, diagnosis, and treatment.
2. *Education*: Chaplains have been found to educate members of the community by interfacing between cultural/religious traditions and healthcare. They train community religious leaders, clergy, seminarians, and so forth, and develop a congregational health ministry for appropriate community orientation.^12^, ^13^, ^14^ This orientation will further empower the members of the local community and their leaders to promote advocacy against the propagation of cancer myths and misconceptions within the community. This will therefore limit late cancer presentations in hospitals.
3. *Advocacy*: Chaplains play numerous roles in health advocacy. Chaplains champion advocacy of patients' psychosocial and spiritual needs within the interdisciplinary healthcare team.^68^ Reports also indicate that chaplains provide many community services including leadership, community participation, community supports, crisis management, and so forth,^12^, ^13^, ^14^ which enable them to function in community healthcare advocacy. They serve as cultural brokers between the healthcare institutions, the patients, and the community by ensuring social justice and patients' rights for spiritual support.^69^ Through community healthcare advocacy and improved spiritual support, myths regarding cancer can be dispelled thereby improving health‐seeking behavior among the people.
4. *Psycho‐social support*: Following a cancer diagnosis, many patients suffer physical, mental, and spiritual distress. This distress can manifest in the form of loneliness, isolation, addiction, and so forth.^70^ Chaplains are skilled listeners who give patients room to tell their stories and provide them with adequate psycho‐social support for proper understanding of their diagnosis especially when patients are in distress. They may also refer to mental health professionals where necessary. This role has been well documented in various reports^70^, ^71^, ^72^ and it is vital in dispelling cancer myths.

### 6.1 Challenges

Spiritual healing as a form of alternative treatment to reduce anxiety, achieve well‐being, and improve overall patient condition has been well documented^73^, ^74^ but its integration and progression in health for holistic and total human care despite World Health Organizations' (WHO) recommendation have been confronted with barriers from the public, healthcare practitioners, policymakers, and so forth.^75^, ^76^ These barriers include but are not limited to spiritual care ambiguity, inadequate training of healthcare providers, limited time and resources, and so forth^77^, ^78^ leading to the noncomprehensive provision of chaplain care. These challenges are described below.

#### 6.1.1 Spiritual care ambiguity

Over the years, authors have struggled to agree on a general definition for the term “spirituality” despite forming a significant part of the patient's experience.^79^ This lack of clarity poses challenges to clinicians who intend to meet the spiritual needs of their patients.^80^ In fact, many individuals, including clinicians, have misconceived spiritual care as a form of religious care due to unfamiliarity with the concepts.^81^, ^82^

Most people also believe that spiritual health practitioners represent a particular faith group thereby limiting the acceptance of spiritual care especially during life‐threatening situations.^83^ This conceptualization of chaplains questions the scope of their practice in many healthcare settings. Some chaplains are rejected in some healthcare settings due to these misconceptions. Hence, chaplains are seen as a minority group in the healthcare system and their inclusion may only be based on the physician's discretion due to these conceptual ambiguities. The case is even worse in Nigeria due to the nonexistence of chaplains in most Nigerian hospitals.

Similarly, challenges in identifying patient's spiritual distress, the form of spiritual care required and the need for referrals on the part of the clinicians have been documented^84^, ^85^ and they detailed inadequate knowledge in spiritual care, role confusion due to conflicting perspectives about spiritual care, overly medical focus, and so forth^82^, ^86^ as barriers leading to these situations. Some clinicians believe that the members of the family must seek spiritual support for their sick relatives while others believe that it is the role of the clinicians to inquire about the patient's spiritual distress. This misunderstanding limits prompt referrals for spiritual care services leaving patients with limited access to necessary chaplain support.^87^

#### 6.1.2 Inadequate training of healthcare providers

Currently, not enough emphasis is placed on spiritual care competencies in healthcare training curricula when compared with the management and technical imperatives involved in healthcare training and practices.^88^ Most medical schools are wary of expanding the curriculum to incorporate spiritual and religious training. This may be due to the limited knowledge of clinicians on the roles of chaplains. Similarly, research has also shown that training of nurses in spiritual care has been associated with inconsistencies including vague teaching and unwarranted simplifications of critical aspects leading to inadequate knowledge.^89^, ^90^

A study on nurses' views about the barriers to spiritual care reported that about 70% of nurses felt they received inadequate training on spirituality which affected their ability to meet their patients' spiritual needs.^91^ Another similar study in Nigeria revealed that about 60% of nurses have no education in spiritual care. In fact, among those who have some knowledge about spiritual care, only about 20% submitted that they got the training as part of their undergraduate curriculum. This underscores the fact that spiritual care receives very low consideration in various healthcare curricula.

In addition, some clinicians believe that family members would be uncomfortable discussing spiritual distress with anyone other than their spiritual care practitioners and they conveniently avoid such discussions. This belief gives limited room for consideration of referrals to chaplains. A lot of times, inquiries about spiritual distress get brushed over because clinicians attach less importance to them. This, therefore, reveals why spiritual care practices among clinicians are suboptimal when compared with their overall clinical duties.^64^ Spirituality rarely receives enough attention in the form of in‐service training or clinical meetings among clinicians^92^ and if this is not properly addressed, it may become a neglected practice in healthcare settings.^93^

In light of this, inadequate clinician training and expertise in offering spiritual care remains a constraining factor in chaplain care. It is quite apparent that clinicians are not adequately equipped in this domain of patient care which calls for concern.

#### 6.1.3 Limited time and resources

Healthcare professionals mostly spend limited time with patients due to hectic working conditions. Hence, may give less priority to the spiritual care of patients.^94^ A study to assess the attitudes of 267 oncology nurses to spirituality revealed that spiritual care ranked among the bottom in terms of priority of patient care. The need to address pain, encourage nutrition, and diagnosis are given preferences.^95^ Patients who find it quite uncomfortable expressing their spirituality may require a long engagement with the clinicians for them to express their needs and this demand may be difficult for healthcare professionals to meet. Therefore, time constraints become a barrier to their spiritual care.^81^, ^96^

Similarly, the lack of spiritual facilities and resources in the hospital is another constraint to chaplain care in many healthcare settings. Musa et al.^94^ also reported the absence of spiritual leaders and lack of resources as the major barriers to the provision of spiritual care in the hospital.

### 6.2 Recommendations

Chaplaincy has been employed in schools, prisons, the military, and so forth^97^, ^98^, ^99^ but its deployment in the Nigerian healthcare system is still undesirable. Chaplains have numerous roles in the Nigerian healthcare system, especially in end‐of‐life care and dispelling myths associated with cancers. A look at the aforementioned roles especially in other climes indicates that they are valuable members of the healthcare team. Therefore, the need to enhance these roles cannot be overemphasized.

First, there is a need to review the various healthcare curriculum in schools. The urgent need to incorporate spiritual care practices in the course work of medical students and nurses to improve their knowledge of spiritual care and make appropriate referrals is pertinent. Also, the roles of spiritual care in patient management should be discussed regularly at clinical meetings to update clinicians on this domain of patient care. In addition, conferences, workshops, and seminars on the various aspects of spiritual care and spirituality should be organized for adequate updates on the roles of spirituality in patient care.

Second, chaplains should be incorporated into the healthcare team, especially in palliative care or hospices. Patients should be able to engage with enough confidence when in need of proper counseling and psycho‐social support. Therefore, chaplains should be employed across all levels of patient care from primary to secondary and tertiary care. This will also enhance their community participation especially when they function in primary healthcare for dispelling cancer misconceptions in the community.

## 7 CONCLUSION

The epidemiological burden of cancer in Nigeria is on the rise over the past two decades due to exposure to numerous risk factors. What compound the problem include various myths and misconceptions. However, due to high religiosity in Nigeria, religious leaders can play crucial roles in dispelling these myths and misconceptions about cancer. This narrative review reveals that myths contribute to the underutilization of cancer prevention and care practice among people with cancer. Hence, improved public awareness about cancer is very imperative. Chaplains' roles include counseling, advocacy, education, and psycho‐social support which may be limited by challenges such as spiritual ambiguity, inadequate recognition of their roles, and limited time/resources. These challenges can be addressed by training and incorporating chaplain practice in cancer care

## AUTHOR CONTRIBUTIONS

**Afeez Abolarinwa Salami**: Conceptualization; data curation; formal analysis; funding acquisition; investigation; methodology; project administration; resources; software; supervision; validation; visualization; writing—original draft; writing—review and editing. **Kehinde Kazeem Kanmodi**: Conceptualization; funding acquisition; project administration; resources; supervision; writing—review and editing. **Jimoh Amzat**: Resources; supervision; writing—review and editing.

## CONFLICT OF INTEREST STATEMENT

Kehinde Kazeem Kanmodi is an Editorial Board member of Health Science Reports and a co‐author of this article. To minimize bias, they were excluded from all editorial decision‐making related to the acceptance of this article for publication. The remaining authors declare no conflict of interest.

## TRANSPARENCY STATEMENT

The corresponding author Kehinde Kazeem Kanmodi affirms that this manuscript is an honest, accurate, and transparent account of the study being reported; that no important aspects of the study have been omitted; and that any discrepancies from the study as planned (and, if relevant, registered) have been explained.

## Data Availability

Data sharing is not applicable to this article as no new data were created or analyzed in this study.
